# Stress and steroid regulation of synaptic transmission: from physiology to pathophysiology

**DOI:** 10.3389/fncel.2012.00069

**Published:** 2013-01-17

**Authors:** Nicola Maggio, Harmen J. Krugers, Menahem Segal

**Affiliations:** ^1^Department of Neurology, The Chaim Sheba Medical CenterTel HaShomer, Israel; ^2^Swammerdam Institute for Life Sciences, University of AmsterdamAmsterdam, Netherlands; ^3^Department of Neurobiology, The Weizmann Institute of ScienceRehovot, Israel

Upon exposure to stressful experiences, steroid hormones, neurotransmitters, and neuromodulators are released which modulate specific processes in the brain. While the release of these compounds is believed to promote behavioral adaptation to stressful experiences, they have also been implicated in stress-related psychopathology. Extensive research in the past decade has culminated in a deeper understanding of the cellular and molecular mechanisms of how stress hormones, neurotransmitters, and neuromodulators, alone and in concert, affect the brain. This new multidisciplinary approach involving behavioral, electrophysiological, molecular, and epigenetic studies is used to elucidate the long-lasting complex effects of stress on cognitive functions in the brain. The target for the action of these mediators ranges from membrane receptors to nuclear receptors, often specific for different brain areas, affecting eventually homeostatic and various cognitive functions.

In this Frontier Research Topic, we have put together chapters written by leaders in the field that provide up-to-date summaries of the different angles of work on the effects of steroid hormones, neurotransmitters, and neuromodulators on synaptic transmission and plasticity from ion channels to pathophysiological processes. The different chapters deal with epigenetics (Hunter, 2012), which details the different nuclear targets for the long-term effects of stress. Mody and Maguire (2012) discuss the role of GABA in the feedback regulation of steroid action, Levy and Tasker summarize the current knowledge on the regulation of the HPA axis (Levy and Tasker, 2012). The main section of the Frontier Topic involves novel views on postsynaptic effects of steroid hormones, CRH, and noradrenaline on synaptic functions in the brain. These include a section on amygdala-hippocampus interaction (Li and Richter-Levin, 2012), cellular, and molecular studies on CRH effects in the hippocampus (Chen et al., 2012), effects of early life stress on metabolic functions in the brain (Bock et al., 2012), interactions between noradrenaline and corticosterone on brain function (Krugers et al., 2012), region selective effects of corticosterone in the hippocampus (Maggio and Segal, 2012), and finally, effects of corticosterone on NMDA receptor function in the hippocampus (Tse et al., 2012). Finally, a behavioral study on the interaction between gestational and adult stress (Walf and Frye, 2012) concludes the list.

Altogether, these papers provide state-of-the-art insights how stress determines cellular and network function and ultimately how stress affects cognition and emotion in the brain, a subject of increasing importance in modern society.

## References

[B1] Bock J. Riedel A. Braun K. (2012). Differential changes of metabolic brain activity and interregional functional coupling in prefronto-limbic pathways during different stress conditions: functional imaging in freely behaving rodent pups. Front. Cell. Neurosci. 6:19 10.3389/fncel.2012.00019 22590453 PMC3349270

[B2] Chen Y. Andres A. L. Frotscher M. Baram T. Z. (2012). Tuning synaptic transmission in the hippocampus by stress: the CRH system. Front. Cell. Neurosci. 6:13 10.3389/fncel.2012.00013 22514519 PMC3322336

[B3] Hunter R. G. (2012). Epigenetic effects of stress and corticosteroids in the brain. Front. Cell. Neurosci. 6:18 10.3389/fncel.2012.00018 22529779 PMC3329877

[B4] Krugers H. J. Karst H. Joels M. (2012). Interactions between noradrenaline and corticosteroids in the brain: from electrical activity to cognitive performance. Front. Cell. Neurosci. 6:15 10.3389/fncel.2012.00015 22509154 PMC3321636

[B5] Levy B. H. Tasker J. G. (2012). Synaptic regulation of the hypothalamic–pituitary–adrenal axis and its modulation by glucocorticoids and stress. Front. Cell. Neurosci. 6:24 10.3389/fncel.2012.00024 22593735 PMC3349941

[B6] Li Z. Richter-Levin G. (2012). Stimulus intensity-dependent modulations of hippocampal long-term potentiation by basolateral amygdala priming. Front. Cell. Neurosci. 6:21 10.3389/fncel.2012.00021 22586371 PMC3343647

[B7] Maggio N. Segal M. (2012). Steroid modulation of hippocam-pal plasticity: switching between cognitive and emotional memories. Front. Cell. Neurosci. 6:12 10.3389/fncel.2012.00012 22454617 PMC3308347

[B8] Mody I. Maguire J. (2012). The reciprocal regulation of stress hormones and GABAA receptors. Front. Cell. Neurosci. 6:4 10.3389/fncel.2012.00004 22319473 PMC3268361

[B9] Tse Y. Bagot R. C. Wong T. (2012). Dynamic regulation of NMDAR function in the adult brain by the stress hormone corticosterone. Front. Cell. Neurosci. 6:9 10.3389/fncel.2012.00009 22408607 PMC3294281

[B10] Walf A. A. Frye C. A. (2012). Gestational or acute restraint in adulthood reduces levels of 5α-reduced testosterone metabolites in the hippocampus and produces behavioral inhibition of adult male rats. Front. Cell. Neurosci. 6:40 10.3389/fncel.2012.00040 23264760 PMC3524518

